# Dataset on the deposits of the semi-circular rampart around the former Viking settlement Hedeby and its vicinity

**DOI:** 10.1016/j.dib.2020.106493

**Published:** 2022-11-04

**Authors:** Anastasiia Kurgaeva, Svetlana Khamnueva-Wendt, Jann Wendt, Hans-Rudolf Bork

**Affiliations:** a Institute of Environmental and Agricultural Biology (X-BIO), Tyumen State University, Semakova str. 10, Tyumen 625003, Russia; b Institute for Ecosystem Research, Christian-Albrechts-University of Kiel, Olshausenstrasse 75, Kiel 24118, Germany

**Keywords:** Hedeby, Rampart, Earthwork, Viking age, Geoarchaeology, Physico-chemical analysis

## Abstract

Soils and sediments are able to preserve traces of human activity in the form of morphological, geochemical and geophysical properties of materials. Thanks to that the study of these materials may provide valuable information about the formation and functioning of archaeological sites. Materials transported for earthwork construction and their configuration preserve important information on the past landscape development, the anthropogenic transformation of the landscape as well as the process of the fortification formation.

The UNESCO heritage site Hedeby was one of the most significant proto-towns in Northern Europe and an important trading center in the Viking Age. The town was surrounded by the semi-circular fortification rampart connected to the Danevirke, the Danish fortification system. Due to its dimensions (maximum height 10 m) and good preservation state, the semi-circular rampart is one of the most prominent features of the area.

In this article the data on the physico-chemical analysis of the materials from the cores along a coring transect across the semi-circular rampart are presented. The following properties were determined: pH, weight percentage of gravel, charcoal, artefacts, and bones, loss on ignition, magnetic susceptibility, grain size distribution ≤2 mm, elemental concentrations.

The data is valuable for geoarchaeological analysis of the landscape transformation and the earthwork construction at Hedeby including the reconstruction of the process and the techniques used in the Viking Age. Data on the buried soil found underneath the rampart deposits might provide insight into the surface soils characteristics prior to the rampart construction.

## Specifications Table

**Table:** 

Subject	Soil science, Earth and Planetary Sciences, Archaeology
Specific subject area	Geoarchaeology, Paleopedology, Paleoenvironmental Reconstruction, Geo-Ecology, Anthropogenic Transformation of Landscape
Type of data	Tables, Figures, Images
How data were acquired	Vibracore corings and Pürckhauer corings were performed along the transect across the semi-circular rampart, which surrounds the Viking settlement Hedeby. The Pürckhauer cores were described and sampled selectively in the field. In the laboratory the cores obtained with the Vibracore system were opened and described, from which three main cores were sampled continuously and analyzed. Dried material was sieved through the sieves of 2 mm mesh size; gravel, charcoal, artefacts and bones were separated, their weight percentages were calculated according to the total weight of the sample. Magnetic susceptibility was measured on a Bartington Instruments MS2B meter and the data was calibrated with the use of the calibration sample of 1% Fe_3_O_4_. Loss on ignition measurements were performed at 550 °C to estimate organic matter content and at 950 °C to estimate carbonate content. The potential pH values were obtained by the potentiometric method in suspension. Grain size distribution ≤2 mm was determined on a Mastersizer 2000 (the laser granulometry method). Element concentrations were measured with the use of the X-ray fluorescence method on a Thermo Scientific Nitron XL3 Analyzer. All analyzes were performed in the laboratories of Kiel University, Germany.
Data format	Raw
Parameters for data collection	Main transect units such as moat, outer slope, rampart surface (two main cores – at its outer and inner side), inner slope, foot of the rampart inside and outside of the settlement were presented by the Vibracore corings. The Pürckhauer corings were performed on the fields on both sides of the rampart to support the main cores. The cores were divided into layers according to morphological properties of the materials. Three main cores (two on the rampart and one in the moat) were sampled continuously: every layer was represented by at least one sample. In addition, several samples were taken from the supporting cores. In case of strong heterogeneity of the materials with clearly seen sublayers, every sublayer was sampled. Homogeneous layers were sampled in their middle part. If a homogeneous layer was thicker than 25 cm, several samples were taken. In the laboratory, basic physico-chemical properties, grain size distribution ≤2 mm, and elemental concentrations of all samples were determined.
Description of data collection	A total of 139 samples were collected along the transect. Two cores representing the entire thickness of the rampart and one core representing the moat filling were sampled continuously. Three main analyzed cores and the supporting cores were described. All collected samples were analyzed in the laboratory.
Data source location	The cores were taken along the transect across the south-western part of the semi-circular rampart of the Viking settlement Hedeby, south of the city of Schleswig, Schleswig-Holstein, Germany. The geographical coordinates of the study area are 54°29′28’’N and 9°33′55’’E.
Data accessibility	Repository name: Mendeley Data Data identification number: https:/doi.org/10.17632/pwjh8gpz93.1 Direct URL to data: https://data.mendeley.com/datasets/pwjh8gpz93/1

## Value of the Data

• The data are valuable for the reconstruction of the construction process of the rampart around the former Viking settlement Hedeby, which is included in the UNESCO Heritage list.
• The data are beneficial for geoarchaeologists, archaeologists and earth science researchers who analyze the earthwork constructions.
• The data give information on the materials that were used for the rampart construction, which in turn indicates the techniques that were applied. This knowledge is relevant for the investigation of other (contemporary) earthworks.
• The data give information on the anthropogenic transformation of the landscape in the Viking Age.
• The data on the soil profile buried under the rampart give an insight on the surface soil existed prior to rampart construction that is useful for the study of the soil genesis and evolution as well as for the environmental reconstructions.

## 1 Data Description

**Fig. 1:**
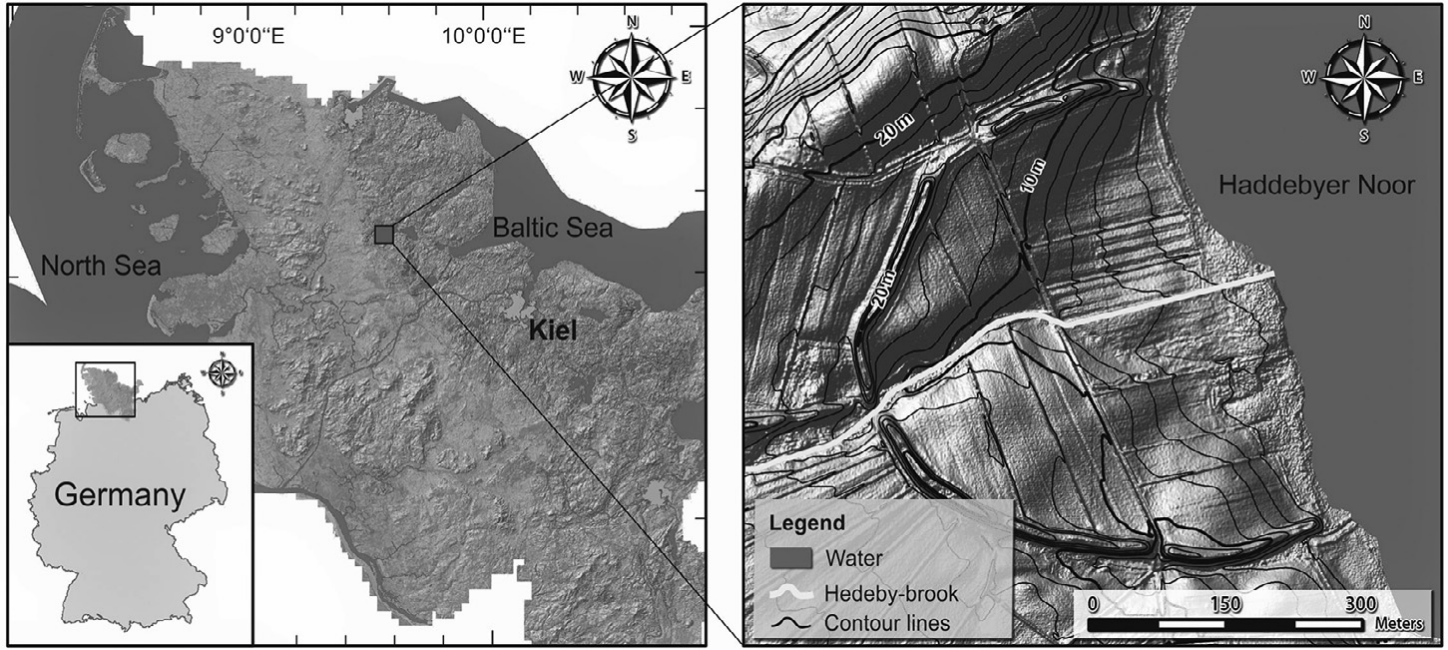
The location of the study area [1].

The study area is located at the former Viking settlement Hedeby, at the western shore of Haddebyer Noor and south of the city of Schleswig, Schleswig-Holstein, Germany. The location of the study object can be seen in Fig. 1. The geographical coordinates of the study area are 54°29′28’’N and 9°33′55’’E.

A series of corings was performed along the transect across the rampart at the western margin of the settlement, around 40 m south of the rampart opening. Seven vibracore and nine Pürckhauer corings were performed starting from 18.3 m east of the rampart's foot inside the settlement and 20.2 m west of the rampart's foot outside the settlement. The rampart itself has the width of 24.1 m at its foot. Therefore, the length of the transect is 62.6 m. The transect reveals a complex structure of anthropogenic genesis with positive (rampart itself) and negative landforms at the both sides of the rampart.

**Fig. 2:**
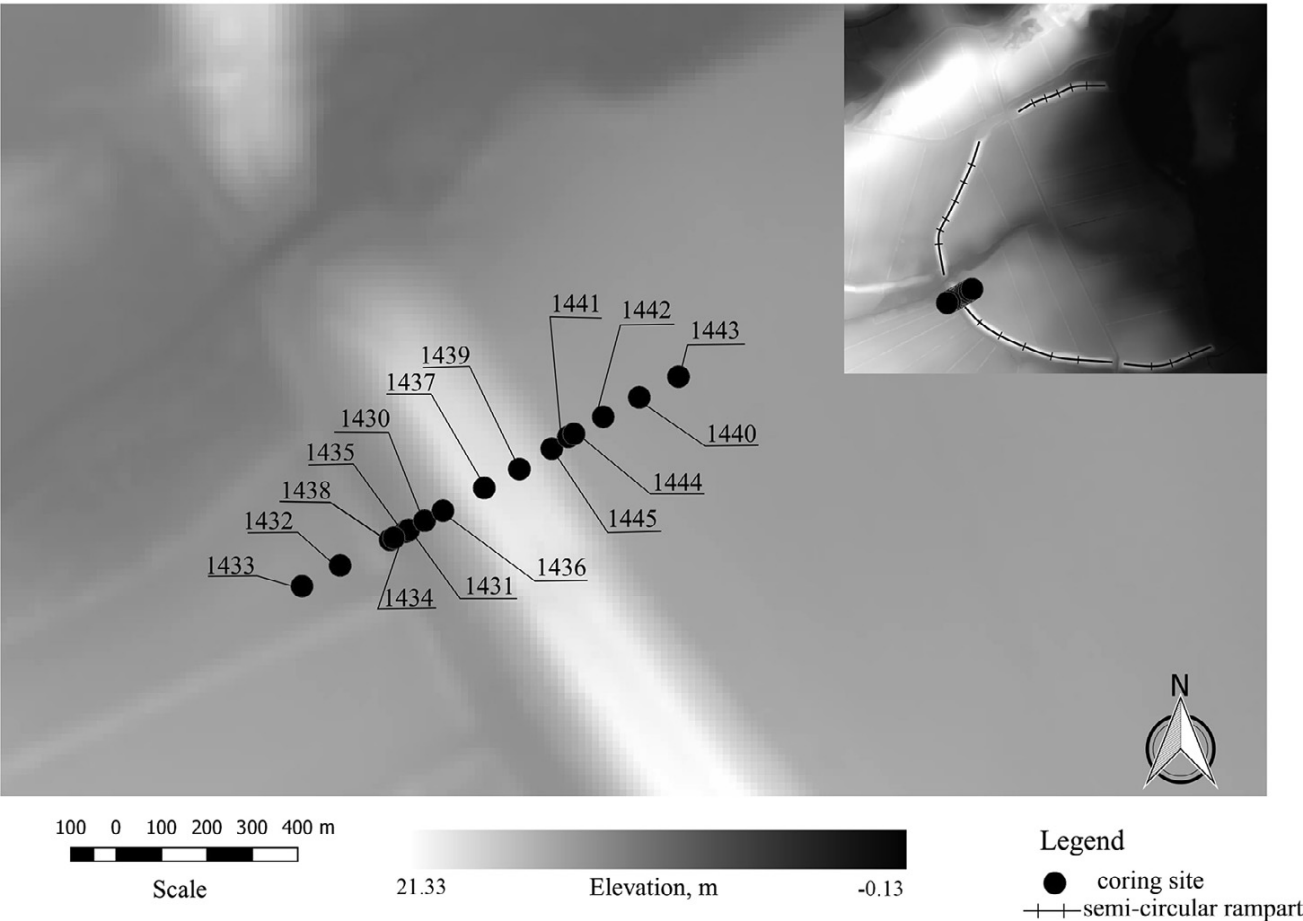
Digital elevation model of Hedeby and its surrounding with the location of the coring sites.

**Fig. 3:**
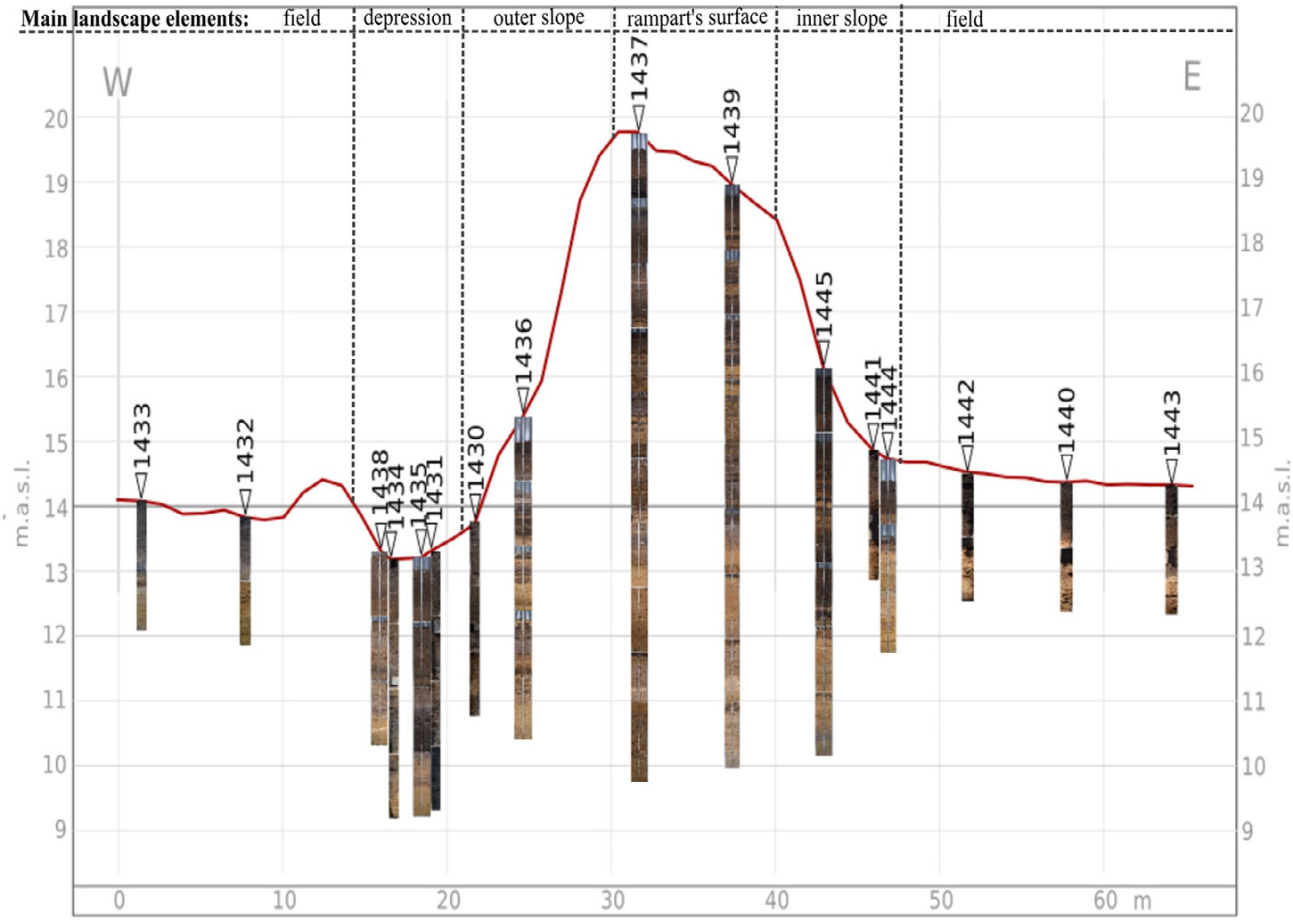
The coring transect across the semi-circular rampart with illustration of the cores.

Fig. 2 shows the location of the coring sites and the relief of the surrounding territory. Fig. 3 represents the overall view of the transect with all cores. The buried soil profile is observed underneath the rampart material at the coring site 1439.

The main landscape elements crossed by the transect are (in direction from west to east): an agricultural field outside the settlement (coring sites 1432, 1433); a hedgerow between the field and the depression (no corings); the depression adjoining the rampart (coring sites 1431, 1434, 1435, 1438); the rampart's outer slope (coring site 1430) with a low plateau (coring site 1436); the rampart's surface (coring sites 1437, 1439); the rampart's inner slope (coring sites 1441, 1444, 1445); a field within the settlement (coring sites 1440, 1442, 1443).

The data on the physico-chemical analyzes are provided in the document “Data on sample analysis_Hedeby.xlsx” shared in the Mendeley Data repository (https://data.mendeley.com/datasets/pwjh8gpz93/1). Basic properties of the sediments (pH, weight percentage of gravel, charcoal, bones and artefacts, loss on ignition (LOI), magnetic susceptibility (MS)) as well as grain size distribution (GSD), and elemental concentrations for the samples from the Pürckhauer cores and cores 1435, 1437 and 1439 are given separately. Special code was assigned to every sample. The samples from the Pürckhauer corings received the code “I”, the samples of cores 1435, 1437 and 1439 got the codes “II”, “III” and “IV”, respectively. The count starts from the lowermost sample. The morphological description of the material of the transect is given in the document “Description of the core material_Hedeby.xlsx” shared in the Mendeley Data repository (https://data.mendeley.com/datasets/pwjh8gpz93/1). Table 1 explains the indices taken for the layer designation, which is based on pedological principles for the buried soil horizons, while for other stratigraphic units specific designations were introduced.

**Table 1:** The layer designation.

Index of the layer	Designation
Main indices	
Ah	soil humus A-horizon
B	soil B-horizon
C	Pleistocene sediment
F	filling of the moat
L	layer of the rampart
M	colluvial layer
O	layer dominated by organic matter and undecomposed litter of plant remains
Y	layer of anthropogenic origin
Additional indices (not applicable for F and L layers)	
clay	material of clay texture
gr	material dominated by gravel
loam	material of loamy texture
sand	material of sandy texture
silt	material of silty texture
b	buried material
ch	material with presence of charcoal
cult	material with inclusion of artefacts and/or bones
layered	material with signs of layered deposition
trans	material with transitional features (or of transitional character)
w	material eroded from the rampart (for colluvial layers)

## 2 Experimental Design, Materials and Methods

### 2.1 Field methods

The field work took place in March and April 2018. The coring transect was performed across the western part of the rampart. It consisted of 7 deep Vibracore corings and 9 Pürckhauer corings.

Pürckhauer coring system consists of a steel auger of 1 m length and ca. 1.6 cm inner diameter with a head and extensions of the same length but with a slightly smaller diameter and additional metal rods for them. This system is manual and a 4 kg nylon hammer is used to insert the auger into the ground. The Pürckhauer cores were described, sampled if needed and photographed in the field.

Vibracore is a mechanical coring system that consists of a handheld impact hammer (23 kg jackhammer), steel augers with ca. 63 mm inner diameter, steel extension rods, and a hydraulic pulling equipment (8 t pulling force) with a bull clump. The vibracore system allows to extract the cores in plastic inner liners (50 mm outer diameter) that are placed into the auger before coring. In the laboratory the plastic tubes with the sediments were opened with a vibration saw.

All extracted cores were photographed, layers and horizons were distinguished and described. The depth, color by Munsell soil color chart [2], texture [3], inclusions in every layer and horizon were documented.

The texture of the Pürckhauer cores was described according to the guidelines in [3], while the texture of the Vibracore corings was described based on the dominance of particles of a certain size. In the description of the core material (“Description of the core material_Hedeby.xlsx” shared in the Mendeley Data repository (https://data.mendeley.com/datasets/pwjh8gpz93/1)), “and” between the texture classes signifies that the material consists of these texture classes of approximately similar proportions, while “+” is used to describe the dominance of the first component in the presence of other components.

The accurately measured coordinates of the coring sites were obtained by a differential GPS system of Leica GNSS smart antenna.

### 2.2 Laboratory methods

The main cores were used for the laboratory analyzes: one central core of the moat (coring site 1435) and two cores of the rampart: at the outer side (coring site 1437) and at the inner side (coring site 1439) of the rampart. 139 samples were taken from the main cores and some Pürckhauer cores. The cores were sampled continuously: every distinguished layer is represented at least by one sample. If the layer was too heterogeneous and additional sublayers were clearly observable, then the samples were taken from every sublayer. In case of the thick homogeneous layers, the samples were taken from the middle part. In specific cases of the thick layers (more than around 25 cm) several samples were taken per layer.

Sample preparation procedure consisted of drying the materials at 40 °C and consequent sieving of the materials through the sieves of 2 mm mesh size to separate the coarse material such as stones (>2 mm diameter) and the fine material such as sand, silt, and clay (≤2 mm diameter). Charcoal pieces, organic remains, artefacts, and bones were extracted. All separated materials of different types were weighted and calculated as a percentage of the total sample mass.

In order to measure MS, the dry material was placed into 10 ml tubes (3 replications) and measured at low frequency (0.46 kHz) with the use of a Bartington Instruments MS2B meter. The calibration was performed by the calibration sample of 1% Fe_3_O_4_ to eliminate the influence of variable conditions during measurements (MS of the calibration sample is 423×10^−5^CGS). Volume-specific MS was measured at 10^−5^*CGS units, which was converted into mass-specific SI units (10^−8^m^3^/kg) [4].

LOI technique was used to estimate organic matter and carbonate contents in the sample. The material of 5 g of the air-dried sample of the fine-earth was placed into ceramic crucibles that stayed in an oven at 105 °C overnight. Afterwards, the crucibles were placed into a muffle furnace at 550 °C for 2 h to burn organic matter and later at 950 °C for 2 h to eliminate carbonates. After every step the crucibles with heated samples were weighted. In order to prevent absorption of air humidity by the heated samples desiccators were used during the cooling phase. The organic matter and carbonate contents equal the loss of the material during heating at 550 °C and at 950 °C, respectively. They were recalculated in percentages from the sample weight after 105 °C heating.

The potential pH values were obtained during the laboratory work. Calcium chloride (0.01 M CaCl_2_) helps extracting hydrogen ions from the solid material into the solution and all available hydrogen ions are measured by the potentiometric method, which is based on the potential measurement between the glass and reference electrodes [5]. 10 g of the sample were placed in a plastic vessel and 50 ml of CaCl_2_ were added according to 1:5 ratio. Afterwards, the samples were shaken for 5 min and then left for 2 h for the hydrogen ion extraction to take place. After this period, pH values were measured in the suspension.

The sample mass taken for GSD measurement depended on its texture: 0.7–0.8 g of the sandy material, 0.5–0.6 g of the silty material, and 0.4–0.5 g of the clayish material. Sample preparation for GSD measuring consisted of the following steps. First, organic matter was destroyed with 10 ml of 35% hydrogen peroxide (H_2_O_2_). In order to accelerate the reaction the samples were placed into a water bath at 60 °C. Hydrogen peroxide was added until no reaction took place. Afterwards, 0.5 ml of tetrasodium pyrophosphate (Na_4_P_2_O_7_) was used as an anticoagulation agent and added to the samples washed from peroxide. The samples were shaken overhead for at least 16 h. The carbonated samples had an additional treatment of 0.5 M sodium acetate buffer solution (CH₃COONa) and stayed at the water bath for 2 days. They were considered carbonate-free when pH of the solution was less than 5 and conductivity after water washing was less than 400 μS/cm. The prepared samples were used for GSD measurement at a Mastersizer 2000 at the Geography Department of Kiel University, where the size of particles was measured with a laser. The output of the measurement was the relative distribution of the volume of particles in a range from 0.02 to 2000 μm for 70 particle diameter intervals [6]. Later the data was recalculated as a volume percentage of clay (0–2 μm), silt (>2–62.5 μm), fine sand (>62.5–200 μm), medium sand (>200–630 μm), and coarse sand (>630–2000 μm) particles [3].

The X-ray fluorescence (XRF) method was used for the quantitative analysis of elements with use of a Thermo Scientific Nitron XL3 Analyzer. Sample preparation consisted of sample grinding by the use of an agate disk mill to homogenize the material. This material was placed into plastic tubes and covered with a 4 µm thick polypropylene foil to facilitate the X-ray injection into the material. In order to standardize the measurement a metal standard (metal disk) and a soil standard with already known element composition were used and their chemical composition was measured by the device. The measurements obtained in mining mode during 300 s interval. The Helium purge was used for the correct detection of light elements such as Mg, Al, Si, and P [7].

## CRediT Author Statement

**Anastasiia Kurgaeva**: Conceptualization, Formal analysis, Investigation, Writing - Original Draft, Visualization. **Svetlana Khamnueva-Wendt**: Conceptualization, Data Curation, Writing - Review & Editing, Visualization, Supervision. **Jann Wendt**: Investigation, Visualization. **Hans-Rudolf Bork**: Writing - Review & Editing, Supervision.

## Declaration of Competing Interest

The authors declare that they have no known competing financial interests or personal relationships which have, or could be perceived to have, influenced the work reported in this article.

## References

[1] Khamnueva-Wendt S., Mitusov A.V., Wendt J., Bork H.R. (2020). Classification of buried soils, cultural, and colluvial deposits in the Viking town Hedeby. Geoarchaeol. Int. J..

[2] A.H. Munsell, Munsell soil color charts, Munsell Color Company, 2000.

[3] (2006).

[4] Dearing J. (1999).

[5] Pansu M., Gautheyrou J. (2006).

[6] (2007).

[7] XL3 Analyzer Version 7.0.1. User's Guide, Thermo Fisher Scientific Niton Analyzers (revision C), November 2010.

